# Effect of different pellet lengths and diameters on growth performance and intestinal function of young pigeons

**DOI:** 10.1016/j.psj.2026.106794

**Published:** 2026-03-15

**Authors:** Kang Cheng, Chenrui Hou, Xuelei Zhang, Hongyue Zhao, Jinrong Wang

**Affiliations:** a School of Biological Engineering, Henan University of Technology, Zhengzhou 450001, China; b School of International Education, Henan University of Technology, Zhengzhou 450001, China

**Keywords:** Pellet length, Pellet diameter, Growth performance, Intestinal function, Young pigeons

## Abstract

Manipulating pellet size provides a potential approach to improving performance in poultry. However, research is limited on the appropriate pellet size required by young pigeons. Thus, effect of pellet length and diameter on growth performance and intestinal function of young pigeons was investigated in two experiments. In Experiment 1, 150 two-month-old young White King pigeons were fed pellets with 5 (control, CON)-, 7-, and 9-mm length for 3 months, respectively. Compared to the CON, feeding 7- and 9-mm length pellets decreased body weight at 3 months of age (*P* = 0.008), and body weight at 4 months of age (*P* = 0.031) was inhibited by 9-mm length pellets. Interestingly, feed-to-gain ratio of young pigeons fed pellets of 7-mm length during 4 to 5 months was lower than that of other 2 groups (*P* = 0.043). In male birds, feeding 9-mm length pellets decreased (*P* = 0.031) duodenal crypt depth (CD) compared to other 2 groups and increased (*P* = 0.021) duodenal villus height (VH):CD ratio compared to the CON; feeding 7-mm length pellets decreased (*P* = 0.040) ileal VH compared to the CON. In female birds, VH in both duodenum (*P* < 0.001) and jejunum (*P* = 0.023) were increased by 9-mm length pellets compared to the CON. Young pigeons given 7- and 9-mm length pellets had lower duodenal maltase activity (female, *P* = 0.014) compared to the CON. Feeding 9-mm length pellets reduced duodenal sucrase activity (*P* = 0.028) and increased ileal maltase (*P* < 0.001) and sucrase (*P* = 0.036) activities in female pigeons compared to 5- and/or 7-mm length pellets. Additionally, ileal sucrase activity in male birds fed 7-mm length pellets was higher (*P* = 0.004) than that in other 2 groups. In Experiment 2, 150 two-month-old young White King pigeons were fed pellets with 2 (CON)-, 3-, and 4-mm diameter for 3 months, respectively. Increased diameter pellets enhanced VH (*P* = 0.002) and VH:CD ratio (*P* = 0.004), and reduced CD (*P* = 0.017) in duodenum of male pigeons compared to the CON. In contrary, jejunal CD (*P* = 0.010) in female birds was increased and ileal maltase (*P* = 0.038) activity in male birds was decreased by larger diameter pellets. Supplemental 3-mm diameter pellets increased jejunal sucrase activity in male birds compared to those fed other 2 diameters pellets (*P* = 0.016). We concluded that young pigeons show a preference for shorter length pellets during 2 to 3 months of age, and feeding them longer length pellets at the later stages can improve their intestinal function and compensate for their growth. Furthermore, gender differences were observed in the impact of pellet diameters on intestinal function. These results suggest that, to improve young pigeons performance, it is necessary to produce pellets of different sizes according to their growth stage and gender.

## Introduction

In China, meat pigeons have become the fourth most popular poultry for consumption after chickens, ducks, and geese (Ji et al., 2022). It is estimated that approximately 100 million squabs and 60 million pairs of breeding pigeons will be produced in China by 2025. China is the largest producer of meat pigeons in the world, accounting for around 80% of global production (Jiang et al., 2019). In young pigeons, 2 to 5 months of age is considered to be an important growth period. Young pigeons are usually used for reserve breeding. However, young pigeons are very susceptible to different stressors owing to immature development of organs, including their intestines (Sun et al., 2023). The current study focuses on how growth performance, organ development, and intestinal function of young pigeons are affected by the length and diameter of pellets.

Studies on broilers have shown that the physical structure of feed influences the growth performance, nutrient substance utilization, tissues development (e.g., the digestive tract), and health status (Abdollahi et al., 2013; Alsaftli, 2020; Novotný et al., 2023; Zang et al., 2009). Zang et al. (2009) reported that feed pellets enhanced growth performance in broiler chickens during the starter, grower and overall periods by improving nutrient metabolizability and digestive tract development. Additionally, it has been reported that crumble-pellet improved growth performance in broilers throughout the entire period with the effects being more evident during the starter and grower phases compared to mash diets, and the effects of feed particle size varied depending on feed form (Lv et al., 2015). In broiler industry, it is widely recognized that the physical quality of pellets is important in improving growth responses (Corzo et al., 2011; Svihus et al., 2024a, 2024b). Birds can choose the appropriate pellet size according to variation of their oral cavity and break with age. The recommendation concerning the ideal size of pellets varies depending on the growth period. Moreover, altering the size of pellets (length and diameter) can significantly affect their quality and performance of broilers (Abdollahi et al., 2013; Alsaftli, 2020). Thus, manipulating pellet size (in terms of diameter and length) may provide a potential approach to improving performance in birds based on the growth period.

Although the majority of young pigeons are fed pelleted diets, research is limited on the appropriate pellet size (length and diameter) required by young pigeons. The aim of this study was to investigate how pellet size (diameter and length) affected growth performance, organ index, intestinal morphology and digestive enzymes activities in young pigeons. The results will provide the theoretical references for the precise processing and production of pellet feed, contributing to the growth, development and health status of young pigeons.

## Materials and methods

### Birds and experimental design

The experimental design and procedures were approved by the Institutional Animal Care and Use Committee of Henan University of Technology (Zhengzhou, China).

In Experiment 1, a total of 150 two-month-old young White King pigeons (half male and half female) were weighed and allocated to three groups. Pigeons were fed pellets with 5 (control)-, 7-, and 9-mm length for 3 months, respectively. The composition and nutrient levels of the diet are presented in Table S1. Corn, sorghum and soybean meal that was ground through a 1- or 2-mm screen blended with all other ingredients to yield a mash diet, and then pelleted using a pellet mill. The desired pellet lengths (5, 7, and 9 mm) with a similar diameter were achieved by changing the distance between the knives and the same pellet die. In each group, pigeons were assigned in five flying sheds (ten pigeons in each, five male and five female). The dimensions of the flying shed are 3.4 *m* × 1.1 *m* × 3.0 m. During the experiment period, pigeons had free access to feed and water and were housed in a temperature controlled room with natural lighting.

In Experiment 2, a total of 150 two-month-old young White King pigeons (half male and half female) were weighed and allocated to three groups. Pigeons were fed pellets with 2 (control)-, 3-, and 4-mm diameter for 3 months, respectively. The desired pellet diameters (2, 3, and 4 mm) with a similar length were achieved by changing the diameter of die hole without changing the distance between the knives and the pellet die. In each group, pigeons were assigned in five flying sheds (ten pigeons in each, five male and five female). Housing, diet formulation, pellet processing, and management of pigeons were the same as described for Experiment 1.

### Growth performance

At 2, 3, 4, and 5 months of age, body weight (BW) of the pigeons was weighed per replicate. Feed intake of pigeons was recorded daily per replicate. Average daily gain (ADG), average daily feed intake (ADFI), and feed-to-gain ratio were calculated for the months 2-3, 3-4, and 4-5, respectively.

### Sample collection

At 5 months of age, twelve pigeons (six male and six female) per group were randomly selected and weighed. The birds were sacrificed by cervical dislocation. Liver, spleen, kidney, bursa of Fabricius, testicle, ovary, fallopian tube, breast muscle, and gizzard were dissected and weighed, and then the organ index [organ weight (g)/BW (g) × 100%] was calculated. Approximately 2 cm long segments of the mid-duodenum, jejunum and ileum were collected, flushed with physiological saline, and fixed in 4% polyformaldehyde to assess intestinal morphology. The intestine (duodenum, jejunum and ileum) mucosa samples were put into sterile tubes, immediately snap-frozen in liquid nitrogen, and stored at −80°C for subsequent analysis.

### Intestinal morphology analysis

After fixation, duodenum, jejunum and ileum samples were dehydrated, cleared, embedded, sectioned, stained, and photographed under a light microscope (RVL-100-G; ECHO Laboratories, San Diego, CA, USA). In each section, villus height (VH) and crypt depth (CD) were measured using Image-Pro Plus 6.0 software (Media Cybernetics, San Diego, CA, USA), and VH:CD ratio was then calculated.

### Intestinal disaccharidase activity assay

The activities of intestinal mucosal lactase, maltase, and sucrase were determined by colorimetric kits (Nanjing Jiancheng Institute of Bioengineering, Nanjing, China).

### Statistical analysis

The SPSS software (ver. 22.0 for Windows, SPSS Inc., Chicago, USA) was used for analyzing experimental results. Significant differences in growth performance at every month, organ index, intestinal morphology and digestive enzyme activity of 5-month-old pigeons between groups were determined by the one-way analysis of variance fitting the pellet size as the only fixed effect, followed by the Tukey’s post hoc test for pairwise comparisons. Results are presented as mean and standard error. A *P* value less than 0.05 was considered statistically significant.

## Results

### Experiment 1

Feeding longer pellets decreased BW at 3 months age (7 and 9 mm, *P* = 0.008) and 4 months of age (9 mm, *P* = 0.031) compared to control birds that fed short (5 mm) pellets (Table 1). Interestingly, feed-to-gain ratio of young pigeons during 4 to 5 months fed pellets of 7-mm length was lower than that of pigeons fed 5- and 9-mm length pellets (*P* = 0.043). There was no difference in ADG and ADFI among 3 groups (*P* > 0.05). Female young pigeons fed pellets of 9-mm length had lower spleen organ index than those given 7-mm length pellets (*P* = 0.007, Table 2). Pellet length did not affect liver, kidney, bursa of Fabricius, testicle, ovary, fallopian tube, breast muscle, and gizzard index of young pigeons (*P* > 0.05).

**Table 1:** Effect of pellet lengths on growth performance in young pigeons.

Items	Pellet lengths (mm)			*P-*value
	5	7	9	
Body weight (g/bird)				
2 month of age	504.40±9.40	494.30±6.40	505.90±6.30	0.492
3 month of age	599.50±12.10^a^	563.80±10.00^b^	557.40±7.80^b^	0.008
4 month of age	607.20±10.10^a^	577.60±11.60^ab^	569.60±10.80^b^	0.031
5 month of age	612.10±10.00	590.10±12.30	583.80±11.20	0.147
Average daily gain (g/bird/d)				
2 to 3 month of age	3.30±0.40	3.00±0.40	2.30±0.50	0.305
3 to 4 month of age	0.30±0.10	0.40±0.10	0.40±0.20	0.389
4 to 5 month of age	0.50±0.10	0.50±0.20	0.20±0.10	0.111
Average daily feed intake (g/bird/d)				
2 to 3 month of age	48.30±0.90	46.50±1.90	49.00±0.70	0.837
3 to 4 month of age	40.50±2.20	37.00±1.90	45.00±2.60	0.408
4 to 5 month of age	40.00±2.50	37.00±2.30	42.00±3.20	0.081
Feed-to-gain ratio				
2 to 3 month of age	15.70±2.10	16.40±2.10	25.90±8.30	0.420
3 to 4 month of age	161.00±22.70	107.80±42.37	84.60±54.30	0.283
4 to 5 month of age	246.10±41.60^a^	82.80±12.90^b^	143.50±47.40^a^	0.043

a-b Means within a row with different superscripts are different at *P* < 0.05. Results are presented as mean and standard error.

**Table 2:** Effect of pellet lengths on organ index in young pigeons (%).

Items	Pellet lengths (mm)						*P-*value	
	Male			Female				
	5	7	9	5	7	9	Male	Female
Liver	1.43±0.21	1.58±0.11	1.62±0.25	1.97±0.39	1.53±0.07	1.59±0.09	0.745	0.331
Spleen	0.07±0.01	0.04±0.01	0.07±0.02	0.05±0.01^ab^	0.09±0.01^a^	0.03±0.01^b^	0.385	0.007
Kidney	0.54±0.06	0.55±0.08	0.51±0.08	0.50±0.03	0.56±0.05	0.44±0.06	0.959	0.291
Bursa of Fabricius	0.03±0.01	0.05±0.02	0.05±0.01	0.05±0.01	0.07±0.01	0.06±0.01	0.897	0.286
Testicle	0.41±0.11	0.58±0.10	0.21±0.14	—	—	—	0.151	—
Ovary	—	—	—	0.65±0.09	0.53±0.07	0.56±0.15	—	0.440
Fallopian tube	—	—	—	1.24±0.09	1.26±0.10	1.45±0.24	—	0.932
gizzard	1.18±0.06	1.27±0.08	1.59±0.20	1.43±0.14	1.58±0.10	1.40±0.12	0.071	0.546
Breast muscle	1.43±0.21	1.58±0.21	1.55±0.35	1.47±0.07	1.45±0.06	1.44±0.04	0.727	0.561

a-b Means within a row with different superscripts are different at *P* < 0.05. Results are presented as mean and standard error.

In male birds (Table 3), feeding 9-mm length pellets decreased (*P* = 0.031) duodenal CD compared to 5- and 7-mm length pellets and increased (*P* = 0.021) duodenal VH:CD ratio compared to 5-mm length pellets; feeding 7-mm length pellets decreased (*P* = 0.040) ileum VH compared to 5-mm length pellets. In female birds, VH in both duodenum (*P* < 0.001) and jejunum (*P* = 0.023) were increased by 9-mm length pellets compared to 5-mm length pellets, and similar result was observed for jejunum CD (*P* = 0.003).

**Table 3:** Effect of pellet lengths on intestinal morphology in young pigeons.

Items	Pellet lengths (mm)						*P-*value	
	Male			Female				
	5	7	9	5	7	9	Male	Female
Duodenum								
Villus height (μm)	794.07±54.34	890.17±23.24	1057.00±105.67	711.45±31.18^b^	664.28±10.61^b^	849.47±2.96^a^	0.159	<0.001
Crypt depth (μm)	136.94±5.74^a^	136.34±2.64^a^	108.75±4.15^b^	140.52±25.32	105.90±6.09	126.72±14.35	0.031	0.373
Villus height: crypt depth ratio	6.20±0.37^b^	7.47±0.46^ab^	9.78±1.35^a^	5.28±1.18	6.30±0.27	7.57±0.60	0.021	0.125
Jejunum								
Villus height (μm)	615.97±48.74	679.37±7.47	753.47±7.17	450.97±14.20^b^	571.36±54.81^ab^	733.45±47.86^a^	0.095	0.023
Crypt depth (μm)	71.80±3.93	81.67±4.24	82.10±5.70	63.10±2.23^b^	75.08±1.55^a^	83.51±2.01^a^	0.346	0.003
Villus height: crypt depth ratio	8.88±0.56	6.18±0.72	9.21±0.56	7.15±0.03	7.51±1.10	8.78±0.48	0.083	0.221
Ileum								
Villus height (μm)	802.85±56.35^a^	575.30±5.90^b^	779.05±30.55^ab^	782.97±13.87	756.67±45.81	680.09±50.45	0.040	0.359
Crypt depth (μm)	92.18±2.85	77.40±6.67	106.73±21.70	84.57±0.50	75.63±4.24	79.67±6.67	0.407	0.565
Villus height: crypt depth ratio	9.22±1.06	7.40±0.90	7.11±1.20	9.26±0.22	10.01±0.30	8.03±0.71	0.366	0.117

a-b Means within a row with different superscripts are different at *P* < 0.05. Results are presented as mean and standard error.

Young pigeons given pellets of 7- and 9-mm length had lower maltase (female, *P* = 0.014) activity in duodenum compared with the 5-mm length pellets (Table 4). Feeding 9-mm length pellets reduced duodenal sucrase activity (*P* = 0.028) and increased ileal maltase (*P* < 0.001) and sucrase (*P* = 0.036) activities in female pigeons compared to 5- and/or 7-mm length pellets. Additionally, ileal sucrase activity (*P* = 0.004) in male birds and duodenal lactase activity (*P* = 0.007) in female birds fed a 7-mm length pellet were higher than that in the 5- and 9-mm length pellets groups. Treatments did not affect jejunal disaccharidase activity of young pigeons (*P* > 0.05).

**Table 4:** Effect of pellet lengths on intestinal digestive enzymes and alkaline phosphatase activities in young pigeons (U/mg protein).

Items	Pellet lengths (mm)						*P-*value	
	Male			Female				
	5	7	9	5	7	9	Male	Female
Duodenum								
Lactase	2.06±0.47	3.50±1.35	4.66±2.85	1.38±0.04^b^	7.15±0.84^a^	2.76±0.05^b^	0.430	0.007
Maltase	15.94±1.56	15.48±4.79	12.82±1.87	34.71±3.77^a^	13.79±2.24^b^	11.46±0.74^b^	0.797	0.014
Sucrase	27.13±6.18	17.34±4.77	14.82±2.08	69.16±7.55^a^	77.43±12.28^a^	18.14±0.04^b^	0.287	0.028
Jejunum								
Lactase	1.18±0.56	1.21±0.27	1.75±0.99	2.10±0.24	4.73±2.59	0.84±0.35	0.797	0.318
Maltase	43.19±13.50	50.24±10.25	44.07±7.90	57.65±10.22	38.76±0.25	37.31±7.16	0.911	0.231
Sucrase	17.78±0.63	7.82±1.62	7.30±3.42	11.21±3.09	6.89±1.37	11.56±0.57	0.073	0.317
Ileum								
Lactase	2.93±0.63	2.76±1.02	3.30±2.23	4.37±0.73	2.06±0.16	5.54±0.74	0.951	0.057
Maltase	17.68±3.67	19.73±0.48	13.98±1.09	14.49±0.28^b^	11.45±0.37^b^	39.18±0.78^a^	0.622	<0.001
Sucrase	27.69±0.33^b^	60.36±3.83^a^	28.91±1.19^b^	12.00±3.98^b^	19.22±0.59^ab^	51.21±9.55^a^	0.004	0.036

a-b Means within a row with different superscripts are different at *P* < 0.05. Results are presented as mean and standard error.

### Experiment 2

The effects of pellet diameters were not significant (*P* > 0.05) for BW, ADG, ADFI, and feed-to-gain ratio (Table 5). Spleen index in male birds fed a 4-mm diameter pellet was lower (*P* = 0.016) than that in male birds fed a 3-mm diameter pellet (Table 6), and the value of which was intermediate in male birds fed a 2-mm diameter pellet (*P* > 0.05). Pellet diameters did not affect liver, kidney, bursa of Fabricius, testicle, ovary, fallopian tube, breast muscle, and gizzard index of young pigeons (*P* > 0.05).

**Table 5:** Effect of pellet diameters on growth performance in young pigeons.

Items	Pellet diameters (mm)			*P-*value
	2	3	4	
Body weight (g/bird)				
2 month of age	497.80±7.60	496.90±11.10	494.70±7.70	0.968
3 month of age	554.80±9.50	573.50±10.60	560.20±10.90	0.428
4 month of age	570.20±8.20	585.80±10.90	568.10±9.60	0.370
5 month of age	575.00±8.40	589.70±10.00	577.70±9.90	0.521
Average daily gain (g/bird/d)				
2 to 3 month of age	2.00±0.10	2.60±0.50	2.20±0.40	0.417
3 to 4 month of age	0.40±0.20	0.40±0.10	0.30±0.10	0.687
4 to 5 month of age	0.20±0.10	0.10±0.10	0.30±0.10	0.167
Average daily feed intake (g/bird/d)				
2 to 3 month of age	48.80±1.00	49.40±1.20	48.40±1.20	0.376
3 to 4 month of age	47.80±1.70	45.80±2.70	43.60±1.10	0.916
4 to 5 month of age	44.20±1.90	46.80±2.80	41.60±1.20	0.344
Feed-to-gain ratio				
2 to 3 month of age	24.80±1.70	21.20±4.00	23.70±3.80	0.239
3 to 4 month of age	251.50±82.10	129.00±28.30	153.90±67.70	0.356
4 to 5 month of age	258.90±49.50	312.50±56.50	200.70±59.40	0.743

Note: Results are presented as mean and standard error.

**Table 6:** Effect of pellet diameters on organ index in young pigeons (%).

Items	Pellet diameters (mm)						*P-*value	
	Male			Female				
	2	3	4	2	3	4	Male	Female
Liver	1.43±0.14	1.58±0.10	1.40±0.12	1.47±0.09	1.80±0.07	1.50±0.11	0.724	0.141
Spleen	0.05±0.01^ab^	0.09±0.02^a^	0.04±0.01^b^	0.06±0.01	0.07±0.02	0.05±0.01	0.016	0.664
Kidney	0.47±0.07	0.45±0.06	0.44±0.04	0.49±0.03	0.46±0.05	0.52±0.03	0.961	0.610
Bursa of Fabricius	0.05±0.01	0.07±0.01	0.06±0.01	0.09±0.03	0.04±0.01	0.05±0.02	0.378	0.333
Testicle	0.65±0.09	0.53±0.07	0.56±0.15	—	—	—	0.740	—
Ovary	—	—	—	0.06±0.02	0.07±0.05	0.08±0.02	—	0.877
Fallopian tube	—	—	—	0.51±0.24	0.23±0.17	0.41±0.28	—	0.792
gizzard	1.24±0.09	1.26±0.10	1.45±0.24	1.34±0.06	1.48±0.41	1.33±0.09	0.725	0.889
Breast muscle	1.53±0.22	1.59±0.15	1.50±0.17	1.46±0.09	1.40±0.06	1.51±0.10	0.951	0.890

a-b Means within a row with different superscripts are different at *P* < 0.05. Results are presented as mean and standard error.

Young male pigeons fed 3- and 4-mm diameter pellets showed higher VH (*P* = 0.002) and VH:CD ratio (*P* = 0.004), as well as lower CD (*P* = 0.017) in duodenum, compared to those fed 2-mm diameter pellets (Table 7). In contrary, jejunal CD (*P* = 0.010) in female birds fed 3- and 4-mm diameter pellets were higher than that in birds fed 2-mm diameter pellets. Duodenal morphology in female birds, jejunal morphology in male birds, and ileal morphology in birds were not altered by pellet diameters (*P* > 0.05).

**Table 7:** Effect of pellet diameters on intestinal morphology in young pigeons.

Items	Pellet diameters (mm)						*P-*value	
	Male			Female				
	2	3	4	2	3	4	Male	Female
Duodenum								
Villus height (μm)	750.64±10.74^b^	928.20±4.10^a^	931.26±18.83^a^	981.31±95.69	1059.98±72.15	980.07±16.54	0.002	0.776
Crypt depth (μm)	139.19±3.79^a^	112.13±2.76^b^	119.49±4.46^b^	124.24±12.78	137.65±2.55	131.40±17.83	0.017	0.716
Villus height: crypt depth ratio	5.40±0.22^b^	8.16±0.30^a^	8.25±0.03^a^	7.14±0.91	7.69±0.39	7.58±0.90	0.004	0.852
Jejunum								
Villus height (μm)	519.30±50.73	751.93±63.23	623.16±32.93	619.09±25.24	710.41±35.80	597.54±53.54	0.056	0.135
Crypt depth (μm)	72.55±3.58	84.17±2.55	85.96±4.85	83.64±1.83^b^	97.67±1.57^a^	100.30±6.43^a^	0.145	0.010
Villus height: crypt depth ratio	7.14±0.35	8.02±0.58	6.81±0.62	7.41±0.34	7.28±0.42	5.95±0.15	0.463	0.088
Ileum								
Villus height (μm)	898.62±108.12	651.59±148.52	567.44±2.74	629.33±99.51	657.36±45.65	573.19±101.92	0.317	0.805
Crypt depth (μm)	98.57±17.37	89.80±5.73	70.34±0.24	70.67±4.30	84.69±5.80	76.07±8.74	0.297	0.374
Villus height: crypt depth ratio	9.21±0.53	7.21±0.05	8.50±0.44	7.12±1.18	7.85±0.81	7.48±0.48	0.081	0.857

a-b Means within a row with different superscripts are different at *P* < 0.05. Results are presented as mean and standard error.

Ileal maltase activity in male birds fed 3- and 4-mm diameter pellets were lower than that in male birds fed 2-mm diameter pellets (*P* = 0.038, Table 8). Supplemental 3-mm diameter pellets increased jejunal sucrase activity in male birds compared to those fed 2- and 4-mm diameter pellets (*P* = 0.016). Small intestinal digestive enzymes activities in female birds were unaffected by pellet diameters (*P* > 0.05).

**Table 8:** Effect of pellet diameters on intestinal digestive enzymes and alkaline phosphatase activities in young pigeons (U/mg protein).

Items	Pellet diameters (mm)						*P-*value	
	Male			Female				
	2	3	4	2	3	4	Male	Female
Duodenum								
Lactase	1.72±0.04	2.14±0.36	4.86±1.05	3.27±0.13	2.47±0.10	3.18±2.80	0.074	0.931
Maltase	10.73±0.90	7.33±0.78	6.67±0.47	9.19±1.26	5.41±0.42	4.28±1.08	0.057	0.077
Sucrase	24.42±4.81	12.11±0.84	18.50±0.58	13.61±2.46	16.39±0.21	17.87±6.14	0.118	0.748
Jejunum								
Lactase	0.08±0.01	0.08±0.01	0.08±0.01	2.48±0.14	2.36±0.42	1.80±0.79	0.615	0.659
Maltase	19.42±2.66	24.91±0.08	22.65±2.68	33.45±1.55	20.03±4.70	29.19±2.69	0.336	0.126
Sucrase	12.19±1.64^b^	28.20±3.10^a^	11.42±0.19^b^	13.83±2.15	13.72±0.77	12.65±1.90	0.016	0.872
Ileum								
Lactase	0.08±0.01	0.07±0.01	0.08±0.01	5.14±1.85	6.64±0.58	3.24±1.45	0.436	0.356
Maltase	27.94±1.13^a^	15.89±0.27^b^	15.84±3.32^b^	29.58±13.01	28.41±1.28	34.86±3.21	0.038	0.832
Sucrase	33.14±13.47	17.84±0.09	25.02±1.09	16.96±5.17	18.66±6.01	29.69±6.66	0.475	0.384

a-b Means within a row with different superscripts are different at *P* < 0.05. Results are presented as mean and standard error.

## Discussion

In the present study, the increase in pellet length from 5 to 9 mm reduced BW of pigeons at 3 and 4 months of age without affecting feed-to-gain ratio. These results showed that pellets with a length greater than 7 mm may be too large for young pigeons in the early growth periods and may not be suitable for their oral cavity. They spend more effort and energy breaking down the large pellets, negatively affecting growth rate. Thus, young pigeons in the early stages of growth (2 to 3 months of age) should be suitable for feeding 5-mm length pellets. Interestingly, BW of pigeons fed with 3 different length pellets at 5 months of age was not altered, and feed-to-gain ratio was lowest among those fed 7-mm length pellets during 4 to 5 months period. As young pigeons grow, their oral cavity changes can adapt to large pellets, thereby exhibiting compensatory growth. In terms of feed-to-gain ratio, young pigeons are more suitable for 7-mm length pellets in the later stage of growth (3 to 5 months of age). Additionally, we found no evidence that the growth performance of young pigeons is affected by pellet diameters. Studies investigating the effect of pellet size on growth performance of pigeons were also scarce. Similar results observed in broilers showed that young broilers aged 7 to 14 days exhibit a preference for shorter pellets (Abdollahi and Ravindran, 2013). Abdollahi et al. also reported that feeding 6-mm length pellets to broilers during the grower period increased BW gain and reduced feed per body-weight gain compared to 3-mm length pellets (Abdollahi et al., 2013).

The intestinal morphology, i.e., VH, CD, and VH:CD ratio, is an important indicator of intestinal health, digestion, and absorption (Cheng et al., 2024). The intestinal villi is where nutrients from the feed are transported into the circulation (Novotný et al., 2023). Longer villi has a greater ability to absorb nutrients (Onderci et al., 2006). A large CD indicates rapid tissue turnover and high energy demand for new tissue, as the crypt is considered as a villus factory (Giannenas et al., 2010). A higher VH:CD ratio is an indication of slower turnover rate of intestinal mucosa, resulting in a lower maintenance requirement of intestinal tissues, and finally leading to greater nutrient digestibility and a higher animal growth rate (Cheng et al., 2025; Zang et al., 2009). However, the influence of pellet length and diameter on intestinal morphology parameters in young pigeons have not been reported yet. Only available data from broilers showed that pellet feed increased VH and VH:CD ratio of small intestine compared to those fed the mash diet (Amerah et al., 2007), and intestinal villi morphologically adapt and respond to feed particle size (Qaisrani et al., 2014). In our study, compared to same sex pigeons fed 5-mm length pellets, the duodenal greater VH:CD ratio and reduced CD in male pigeons as well as the duodenal increased VH, jejunal increased VH and CD in female pigeons fed 9-mm length pellets were observed. These results indicated that longer length pellets may be beneficial for intestinal absorptive capacity and contribute to the improved growth performance of pigeons in the later growth period. Additionally, in this study, we also found that VH, CD and VH:CD ratio in duodenum of male pigeons were positively affected by larger-diameter pellets, while CD in jejunum of female pigeons was increased, which may be attributed to gender differences.

Studies investigating the effect of pellet size on disaccharidase activity was also scarce. Intestinal disaccharidase (lactase, maltase, and sucrase) is the key enzyme of carbohydrate digestion and absorption (Tang et al., 2022). In our study, feeding 7-mm length pellets improved ileal sucrase activity in male pigeons and duodenal lactase activty in female pigeons. Feeding 9-mm length pellets have a promoting effect on ileal disaccharidase in female pigeons, but has the opposite effect on the duodenum. These results still indicated that longer length pellets can improve intestinal digestive function. Interestingly, feeding 3-mm diameter pellets exerted different impact in jejunal sucrase and ileal maltase activities in male pigeons, while small intestinal disaccharidases activities in female pigeons were not affected by pellet diameters. These results, when considered alongside those relating to intestinal morphology, suggested that female pigeons have a higher tolerance to pellet diameters, but male pigeons are more suitable for 3-mm diameter pellets.

## Conclusion

In conclusion, young pigeons aged 2 to 3 months show a preference for 5-mm length pellets, and feeding them longer length pellets in the later stages can improve their intestinal function and compensate for their growth. Increased pellet diameters have no effect on growth performance; however, there are gender differences in its impact on intestinal function. These results suggest that in order to improve young pigeons performance, male and female young pigeons should be kept separately and fed pellets of different lengths and diameters during their various growth stages.

## CRediT authorship contribution statement

**Kang Cheng:** Writing – original draft, Validation, Methodology. **Chenrui Hou:** Investigation, Formal analysis. **Xuelei Zhang:** Methodology. **Hongyue Zhao:** Methodology, Data curation. **Jinrong Wang:** Writing – review & editing, Supervision, Resources, Project administration, Funding acquisition, Data curation, Conceptualization.

## Disclosures

The authors declare that they have no conflict of interest.

## Appendix Supplementary materials

Image, application 1
