# Supplementary material for: Effect of different pellet lengths and diameters on growth performance and intestinal function of young pigeons
Source: Poult Sci. 2026 Mar 15;105(6):106794. doi: 10.1016/j.psj.2026.106794 (PMC13054035; doi:10.1016/j.psj.2026.106794)
Supplement: Supplementary file 1 [file mmc1.docx]

Table S1. Ingredients and nutrient composition of the basal diets

| Ingredients (%) | Contents |
| --- | --- |
| Corn | 71.80 |
| Sorghum | 3.00 |
| Soybean meal | 16.52 |
| Dicalcium phosphate | 1.19 |
| Limestone | 1.85 |
| L-Lysine | 0.10 |
| DL-Methionine | 0.13 |
| Premix^1^ | 1.00 |
| Sodium chloride | 0.30 |
| Zeolite powder | 4.11 |
| Total | 100 |
| Calculated nutrient levels |  |
| Crude protein | 14.00 |
| Metabolic energy (MJ/kg) | 11.80 |
| Calcium | 1.00 |
| Total phosphorous | 0.50 |
| Lysine | 0.70 |
| Methionine | 0.36 |

^1^Premix provided per kilogram of feed: vitamin A, 4000.00 IU; vitamin D3, 1725.00 IU; vitamin E, 24.00 mg; vitamin K3, 1.0 mg; vitamin B12,25.00 mg; vitamin B1, 3.0 mg; riboflavin, 13.0 mg; niacin, 15.00 mg; choline chloride, 200.0 mg; pantothenic acid, 7.50 mg; vitamin B6, 2.00 mg; biotin, 0.12 mg; folic acid, 0.55 mg; Fe, 35.0 mg; Cu, 10.0 mg; Mn, 55.0 mg; Zn, 35.0 mg; I, 0.20 mg; Se, 0.25 mg.
